# Clinical Nomogram for Predicting Survival of Esophageal Cancer Patients after Esophagectomy

**DOI:** 10.1038/srep26684

**Published:** 2016-05-24

**Authors:** Jinlin Cao, Ping Yuan, Luming Wang, Yiqing Wang, Honghai Ma, Xiaoshuai Yuan, Wang Lv, Jian Hu

**Affiliations:** 1 Department of Thoracic Surgery, The first Affiliated Hospital, School of Medicine, Zhejiang University, Hangzhou, China

## Abstract

The aim of this study was to construct an effective clinical nomogram for predicting the survival of esophageal cancer patients after esophagectomy. We identified esophageal cancer patients (*n* = 4,281) who underwent esophagectomy between 1988 and 2007 from the Surveillance, Epidemiology, and End Results (SEER) 18 registries database. Clinically significant parameters for survival were used to construct a nomogram based on Cox regression analyses. The model was validated using bootstrap resampling and a Chinese cohort (*n* = 145). A total of 4,109 patients from the SEER database were included for analysis. The multivariate analyses showed that the factors of age, race, histology, tumor site, tumor size, grade and depth of invasion, and the numbers of metastases and retrieved nodes were independent prognostic factors. All of these factors were selected into the nomogram. The nomogram showed a clear prognostic superiority over the seventh AJCC-TNM classification (C-index: SEER cohort, 0.716 vs 0.693, respectively; *P* < 0.01; Chinese cohort, 0.699 vs 0.680, respectively; *P* < 0.01). Calibration of the nomogram predicted the probabilities of 3- and 5-year survival, which corresponded closely with the actual survival rates. This novel prognostic model may improve clinicians’ abilities to predict individualized survival and to make treatment recommendations.

Esophageal cancer is the sixth leading cause of cancer-related death worldwide, and it is responsible for more than 400,000 deaths each year^1^. The mainstay treatment for esophageal cancer without distant metastasis is esophagectomy. For local advanced esophageal cancer, multimodal therapy has been developed that combines preoperative and/or postoperative adjuvant treatment^2^,^3^,^4^. Therefore, accurate prognostic models for predicting survival in patients would be greatly beneficial for counseling and for selection of appropriate treatment strategies.

In the seventh edition (2010) of the American Joint Committee on Cancer (AJCC), a tumor node metastasis (TNM) classification system has been well established. In this classification system, patients with esophageal cancer are stratified according to depth of invasion (T category), number of metastasis nodes (N category), presence of distant metastases, grade, location and tumor histology^5^. This system is the most widely used for prognostic estimates and clinical treatments in patients with cancer. However, some researchers have pointed out that the new system cannot satisfactorily predict the survival of patients with resected esophageal cancer, and is significantly influenced by the number of lymph nodes retrieved and is susceptible to stage migration^6^,^7^. It is believed that other clinicopathological factors such as race, age, tumor size and the number of examined lymph nodes may also influence the patients’ outcomes^8^,^9^.

Recently, clinical nomograms have been developed with intuitive graphs to quantify risk by incorporating important factors for oncology prognostics. Several authors have reported that nomograms are more accurate in prediction and prognosis than the traditional staging systems for patients with colon, breast, gastric, intrahepatic-cholangio or non–small-cell lung cancers^10^,^11^,^12^,^13^,^14^. Until now, however, clinical nomograms for predicting the survival of esophageal cancer patients after esophagectomy have rarely been used.

In this study, we used the Surveillance, Epidemiology, and End Results (SEER) database to construct a clinical nomogram for esophageal cancer patients after esophagectomy, and compared its prognostic value with that of the seventh edition of the AJCC-TNM classification. The SEER database is a population-based cancer database, covers approximately 28% of the U.S. population. However, the SEER cancer registry did not provide information on whether patients were treated with chemotherapy. Therefore, chemoradiation could not be evaluated as a potential prognostic factor in this nomogram. In addition, we used a separate Chinese cohort to externally validate the nomogram.

## Results

### Patient characteristics

A total of 4,281 eligible patients were identified from the SEER database according to the screening queries. Of these 4,281 patients in the primary database, 172 patients (T4N0M0) could not be assigned accurate staging (stage IIIA or IIIC) according to the seventh edition of the AJCC-TNM classification, and were thus excluded. Therefore, a total of 4,109 patients were included in the training cohort. The median follow-up time was 28 months (range of 3 to 276 months), and the 3- and 5-year survival rates of the entire cohort were 49% and 40%, respectively. For the validation cohort, we researched 145 consecutive patients from an independent Chinese cohort. Their median follow-up time was 45 months (range of 3 to 128 months), and the 3- and 5-year survival rates of the entire cohort were 60% and 47%, respectively. The demographics and tumor characteristics of patients in the training and validation cohorts are summarized in Table 1.

### Independent prognostic factors in the training cohort

After a univariate analysis by the Cox-Regression analysis, data on the variables of age at diagnosis, recorded race, histological type, tumor site and size, grade, T category, N category and the retrieved lymph nodes were entered into the multivariable logistic regression analyses. However, sex and adjuvant radiation therapy prior to surgery were not found to be significant. The multivariate analyses demonstrated that the hazard ratios were significantly higher for the factors of older age, black race, squamous cell carcinoma, location of the center of the esophageal, larger tumor size, advanced grade and depth of invasion, increased number of metastasized lymph nodes and decreased number of retrieved lymph nodes (Table 2).

### Prognostic nomogram for survival

**Figure 1:**
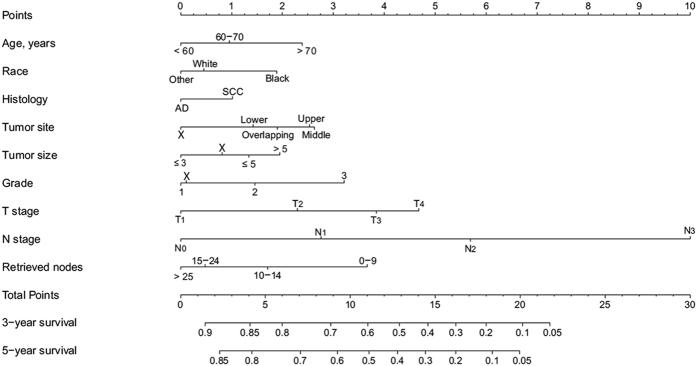
Nomogram predicting 3- and 5-year survival after esophagectomy for esophageal cancer. AD, adenocarcinoma; SCC, squamous cell carcinoma. The X category is used when information on a specific component is unknown.

A nomogram that incorporated all of the significant independent factors for predicting 3- and 5-year survival in the training cohort was established, based on selected variables with hazard ratios (Fig. 1 and Table 2). The nomogram showed that the N category had the largest contribution to prognosis, followed by the T category, retrieved nodes, grade, tumor site, age, tumor size and race. Histological type showed the smallest effect on survival rate. Each variable was given a score on the points scale. By adding up the total scores projected to the bottom scale, we were able to estimate the probability of 3- and 5-year survival.

### Performance of the nomogram

**Figure 2:**
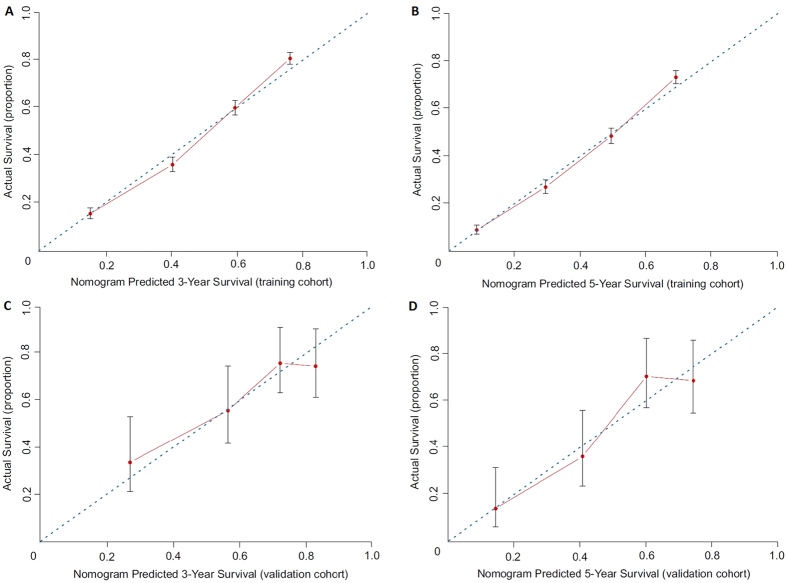
The calibration curves for predicting patient survival at (**A**) 3-year and (**B**) 5-year in the training cohort, and at (**C**) 3-year and (**D**) 5-year in the validation cohort. Nomogram-predicted survival is plotted on the x-axis; actual survival is plotted on the y-axis.

Based on the C-index analysis of the SEER training cohort, the nomogram system provided a better C-index (0.716; 95% CI, 0.706 to 0.726) than the seventh edition of the AJCC-TNM classification (0.693; 95% CI, 0.683 to 0.703; *P* < 0.01). In the Chinese validation cohort, although the patients are drawn from such disparate populations (Table 1), the C-index was also higher for the nomogram system (0.699; 95% CI, 0.641 to 0.757) than for the TNM classification system (0.680; 95% CI, 0.624 to 0.736; *P* < 0.01). A calibration plot of the nomogram is presented in Fig. 2, which shows that the predicted 3- and 5-year survival probabilities for both the SEER training cohort and the Chinese validation cohort agreed well with the actual observations.

**Figure 3:**
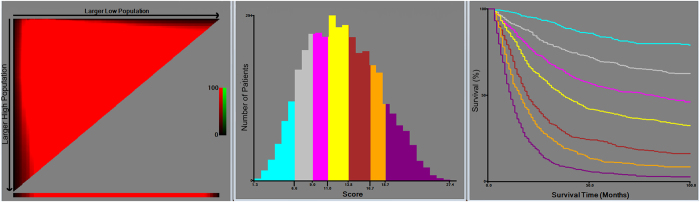
X-tile analysis of survival based on risk scores.

**Figure 4:**
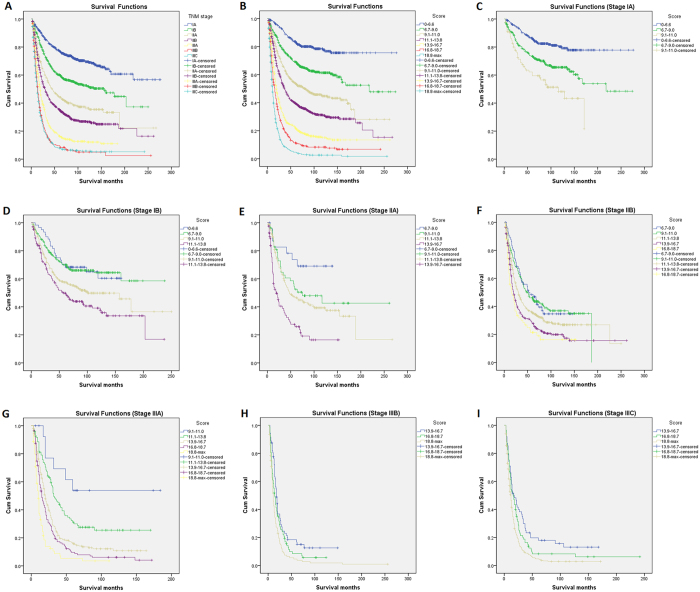
Kaplan-Meier survival curves for patients with esophageal cancer by risk group stratification within each TNM stage ((**A**,**B**), all patients; (**C–I**) stages) in the training cohort. Subgroups with fewer than 12 patients were omitted from the graphs.

According to the optimal cut-off analyses for the SEER training cohort, the cut-off values for total scores were classified as follows: 0 to 6.6, 6.7 to 9.0, 9.1 to 11.0, 11.1 to 13.8, 13.9 to 16.7, 16.8 to 18.7 and ≥18.8 (Fig. 3). The 5-year survival rates were 84%, 69%, 54%, 40%, 22%, 11% and 5%, respectively (*P* < 0.01). For the TNM classification, the 5-year survival rates for stages IA, IB, IIA, IIB, IIIA, IIIB and IIIC were 78%, 60%, 44%, 36%, 16%, 10% and 8%, respectively (*P* < 0.01). Therefore, non-significant distinctions were found for stages IIIB and IIIC (*P* = 0.462). Moreover, within each of the TNM stages, the survival rates predicted by the nomogram showed significant distinctions between Kaplan-Meier curves (Fig. 4).

## Discussion

The AJCC-TNM classification is the most widely used system for prognostic estimates and clinical treatments of patients with cancer. However, this system cannot satisfactorily predict the survival of patients with resected esophageal cancer and is susceptible to stage migration^6^,^7^. Furthermore, our research found that the seventh AJCC-TNM classification could not distinguish the difference in 5-year survival between stages IIIB and IIIC in the training cohort (*P* = 0.462), as the survival of patients with the same stages varied widely. As few studies have developed nomograms for predicting the survival of patients with esophageal cancer, the sample size involved has been small, and the prognostic factors have been limited^15^,^16^,^17^. Thus, we constructed a clinical nomogram to predict the survival of esophagectomy patients based on a large-population-based record system, namely the SEER database of 18 registries.

The SEER database is an authoritative source of information on the incidence of cancer and the survival of patients in the United States. This database covers approximately 28% of the U.S. population. Regarding operative related mortality (which may influence the accuracy of predicting survival), the inclusion criteria of our study were limited to survival of no less than 3 months, according to the most recent report^18^. Through univariable Cox-Regression analysis and subsequent multivariable logistic regression analysis, we identified age, race, histology, tumor site, size, grade, T and N category, and number of retrieved nodes as independent prognostic factors. These findings were consistent with previous reports on survival risk factors for esophageal cancer^8^,^9^,^19^. Finally, a nomogram was constructed for predicting cancer-specific survival. This prediction model confirmed that the TNM factors and four other factors (age, race, tumor size and number of retrieved nodes) were highly related to prognosis. For these four factors, the analyses demonstrated that the risk scores were significantly increased for patients over 70 years old (2.4 points), of black race (1.9 points), having tumor size over 5 cm (1.9 points) and having fewer than ten lymph nodes retrieved (3.7 points).

Notably, our nomogram showed the N category as making the largest contribution to prognosis. The current AJCC-TNM seventh edition has also established the importance of N categories, and has classified N as 0–3 for 0, 1–2, 3–6 and ≥7 nodes, respectively^5^. However, the number of metastasis nodes identified depends significantly on the number of nodes retrieved^7^. As shown in Fig. 1, retrieved nodes were an important prognostic factor for patients with esophageal cancer, and more than 15 nodes reduced the risk scores significantly. Thus, we suggest that the number of retrieved nodes should exceed 15 nodes. Some previous studies have also demonstrated that a higher number of lymph nodes retrieved is associated with better survival^9^,^20^. One possible reason for this finding is that retrieving more lymph nodes makes it more likely that the potentially metastasized lymph nodes will be cleared. Moreover, the number of retrieved nodes may reflect the adequacy of surgical, pathological and institutional care, all of which tend to affect treatment outcomes^20^.

For validation of the nomogram to avoid overfitting and to make sure that the nomogram can be applied generally, it is essential to evaluate discrimination and calibration^13^,^14^,^21^. Discrimination has usually been evaluated with the C-index, and calibration is assessed by comparing the plot of predicted probabilities from the nomogram with that of the actual probabilities. In this study, the discriminative ability of the nomogram showed a clear prognostic superiority over the seventh AJCC-TNM classification, by a C-index of 0.716 vs 0.693 (*P* < 0.01) in the training cohort, and by 0.699 vs 0.680 (*P* < 0.01) in the validation cohort. The calibration plots showed optimal agreement in the training cohort, and acceptable agreement in the validation cohort between the prediction probabilities and actual observations, which ensured the reliability and repeatability of the constructed nomogram. In addition, our analytic approach stratified the total risk score into seven risk groups (using the optimal cut-off analyses in the training cohort), and this approach further demonstrated that the discriminative ability of the nomogram was superior to that of TNM staging. Therefore, stratifying subgroups of patients at different risk levels might make the treatment or design of clinical studies easier for clinicians^14^.

It should be pointed out that neoadjuvant chemoradiation therapy followed by surgery is increasingly applied for patients with resectable esophageal cancer in many centers around the world, although this approach remains investigational^22^,^23^,^24^. In our study, adjuvant radiation therapy prior to surgery was not found to be significant for improving survival. A meta-analysis also showed no clear evidence of a survival advantage with preoperative radiation therapy^25^. Chemoradiation could not be evaluated as a potential prognostic factor in this nomogram. However, a previous study has evaluated neoadjuvant chemoradiation therapy for older patients with esophageal cancer based on the SEER-Medicare database, and this study found that only patients with T4 or lymph node disease appeared to benefit from neoadjuvant chemoradiotherapy^26^.

Our nomogram has several other limitations that are inherent in the SEER database^27^. First, the SEER database is retrospective, the tumor staging is based on pathologic information that is only valid in knowing the outcome for patients after their esophagectomy and with their final pathologic stage in hand, and therefore may introduce the possibility of treatment selection bias. Second, with the advent of modern gene-array technology, some important molecular factors (e.g., VEGF mutation^28^, HER2/neu overexpression^29^) have proven to be predictive of survival. These developments could allow the incorporation of molecular factors to improve this model in the near future.

In conclusion, we have developed and validated a novel nomogram for predicting survival after esophagectomy in esophageal cancer patients. The nomogram is easy to use, and it provides a clear prognostic superiority over the seventh AJCC-TNM staging system. The nomogram might also help clinicians to make individualized predictions of patient survival and to give improved treatment recommendations.

## Methods

### Patient population

We reviewed patient data from the latest version of the SEER as released in September 2015 (covering 18 registries, 1973–2012), by using SEER*Stat version 8.2.1^30^. We extracted cases of patients with microscopically confirmed first invasive esophageal cancer who had received esophagectomies between January 1988 and December 2007. The inclusion criteria were age at diagnosis (codes: 18–85+), histological types (codes: 8000–8576, 8940–8950 and 8980–8981), primary tumor site (codes: 150–155 and 158–159), tumor extension (codes: 10–80 for 1988–2003 and 100–800 for 2004–2007), regional nodes examined (codes:1-90), regional nodes of metastasis (codes: 0-90), survival months (codes: 3-479) and types of follow-up expected (codes: active follow-up). We excluded cases of patients if they had received radiotherapy after surgery, or if data on their tumors with distant metastasis or the distant metastasis were not known. The program selection codes for the SEER database queries are shown in the Supplementary S1. The overall survival estimate registered in the SEER database is the “cause-specific classification of death”, and stratified “dead (attributable to this cancer dx)” or “alive or dead of other cause”. Survival time was calculated from the diagnosis date to the date of death or last contact. The last contact, or the cut-off date of the study, was December 31, 2012, which was the last date of update on the follow-up time. Pathologic staging of patients was characterized according to the seventh AJCC-TNM staging system.

To validate the model, we examined an independent Chinese patient cohort that consisted of 145 consecutive patients with esophageal cancer who had received esophagectomy for esophageal cancer at the first Affiliated Hospital of Zhejiang University between January 2005 and September 2010. All of these patients were selected under the same criteria of inclusions and exclusions that were used in the SEER registries. Moreover, patients who had received preoperative and/or postoperative adjuvant radiotherapy and/or chemotherapy were excluded. The study protocol of the Chinese cohort was performed in accordance with the guidelines outlined in the Declaration of Helsinki and was approved by the Ethics Committee of the first Affiliated Hospital of Zhejiang University. Written informed consent was obtained from all participants.

### Construction of the nomogram

To construct an effective clinical nomogram for esophageal cancer, we developed a model using the SEER database as a training cohort and validated it with an independent Chinese patient cohort. In the training cohort, cancer-specific survival was compared using the univariate Cox-Regression analysis. The significance variables were entered into the multivariable logistic regression analyses using the Cox proportional hazards regression. Finally, using a backward step-down process with an Akaike information criterion (AIC), a nomogram was constructed for predicting 3- and 5-year cancer-specific survival^31^.

### Validation of the nomogram

The prognostic performance of the nomogram was assessed with respect to discrimination and calibration. Discrimination was evaluated with the concordance index (C-index), which was similar to the area under the receiver operating characteristic curve (AUC), but was more appropriate for censored data. The value of the C-index statistic ranged from 0.5 (no discrimination) to 1 (perfect discrimination), and higher C-index values indicated a better prognostic model^32^. Calibration was quantified by comparing the predicted survival with that of the observed survival against the nomogram’s 3- and 5-year predicted cancer-specific survival probabilities. We used the bootstrap technique with 1,000 repetitions for internal validation of the SEER training cohort, and with 200 repetitions for the external validation of the Chinese cohort.

In addition, we conducted a group-stratified analysis of the total risk score to show that the discriminative ability of the nomogram was superior to that of the TNM staging in the training cohort. The optimal cut-off values were determined using X-tile software (http://www.tissuearray.org/rimmlab) and by the minimal *P* value approach^33^.

Statistical analyses were performed using the IBM SPSS statistics version 20.0 (SPSS, Chicago, IL) and R software version 3.1.2 (http://www.r-project.org) with the *rms* and *Hmisc* statistical packages^34^. For all of the analyses, *P* < 0.05 in a two-tailed test was considered to be statistically significant.

## Additional Information

**How to cite this article**: Cao, J. *et al*. Clinical Nomogram for Predicting Survival of Esophageal Cancer Patients after Esophagectomy. *Sci. Rep.* **6**, 26684; doi: 10.1038/srep26684 (2016).

## Supplementary Material

Supplementary Information

## Figures and Tables

**Table 1:** Demographics and Tumor Characteristics of the Surveillance, Epidemiology, and End Results database (training cohort) and Chinese Cohort (validation cohort).

Variables	Training Cohort (N = 4,109)					Validation Cohort (N = 145)		P**
	No. Patients	%	Hazard Ratio	95% CI	P*	No. Patients	%	
Sex					**0.063**			0.009
Male	3366	81.9	Reference			131	90.3	
Female	743	18.1	0.907	0.818–1.005	0.063	14	9.7	
Age, years					**<0.001**			0.874
<60	1570	38.2	Reference			48	33.1	
60–70	1533	37.3	1.112	1.016–1.217	0.021	72	49.7	
>70	1006	24.5	1.367	1.236–1.511	<0.001	25	17.2	
Race					**<0.001**			<0.001
White	3629	88.3	Reference			0	0	
Black	280	6.8	1.352	1.167–1.566	<0.001	0	0	
Other	200	4.9	1.136	0.953–1.353	0.154	145	100	
Histology					**<0.001**			<0.001
Adenocarcinoma	2884	70.2	Reference			7	4.8	
Squamous cell carcinoma§	1225	29.8	1.305	1.201–1.418	<0.001	138	95.2	
Tumor site					**<0.001**			<0.001
Upper	120	2.9	Reference			4	2.8	
Middle	700	17.0	1.102	0.867–1.400	0.428	100	69.0	
Lower	3008	73.2	0.865	0.689–1.085	0.209	41	28.3	
Overlapping	124	3.0	1.080	0.795–1.467	0.622	0	0	
Unknown (X)	157	3.8	0.531	0.383–0.737	<0.001	0	0	
Tumor size (cm)					**<0.001**			0.188
≤3	1240	30.2	Reference			54	37.2	
≤5	1007	24.5	1.804	1.620–2.008	<0.001	30	20.7	
*>*5	807	19.6	2.034	1.818–2.276	<0.001	61	42.1	
Unknown (X)	1055	25.7	1.177	1.053–1.315	0.004	0	0	
Grade					**<0.001**			<0.001
Well differentiated	259	6.3	Reference			13	9.0	
Moderately differentiated	1500	36.5	1.843	1.497–2.269	<0.001	99	68.3	
Poorly or Undifferentiated	1953	47.5	2.761	2.251–3.387	<0.001	33	22.8	
Unknown (X)	397	9.7	1.193	0.932–1.527	0.161	0	0	
Timing of radiation therapy					**0.292**			<0.001
None	2640	64.2	Reference			145	100	
Prior to surgery	1469	35.8	1.044	0.963–1.132	0.292	0	0	
Pathologic T category					**<0.001**			<0.001
T1	1425	34.7	Reference			22	15.2	
T2	778	18.9	1.898	1.683–2.141	<0.001	26	17.9	
T3	1603	39.0	2.868	2.596–3.167	<0.001	93	64.1	
T4	303	7.4	4.777	4.128–5.528	<0.001	4	2.8	
Pathologic N category					**<0.001**			0.968
N0	2355	57.3	Reference			79	54.5	
N1	1009	24.6	2.581	2.353–2.832	<0.001	42	29.0	
N2	511	12.4	3.892	3.479–4.354	<0.001	18	12.4	
N3	234	5.7	5.197	4.479–60.30	<0.001	6	4.1	
Pathologic TNM stage					**<0.001**			0.004
IA	707	17.2	Reference			3	2.1	
IB	666	16.2	1.801	1.503–2.157	<0.001	17	11.7	
IIA	330	8.0	2.835	2.320–3.464	<0.001	21	14.5	
IIB	1053	25.6	3.748	3.201–4.388	<0.001	48	33.1	
IIIA	596	14.5	5.989	5.069–7.076	<0.001	33	22.8	
IIIB	285	6.9	8.430	6.989–10.169	<0.001	13	9.0	
IIIC	472	11.5	8.913	7.512–10.574	<0.001	10	6.9	
No. of nodes retrieved					**0.007**			0.496
0–9	2230	54.3	Reference			73	50.3	
10–14	810	19.7	0.960	0.868–1.063	0.435	37	25.5	
15–24	744	18.1	0.841	0.755–0.937	0.002	29	20.0	
>25	325	7.9	0.861	0.741–0.999	0.049	6	4.1	

^§^According to the 7^th^ edition of the AJCC cancer staging manual, if a tumor is of mixed histopathologic type or is not otherwise specified, it recorded as squamous cell carcinoma.

^*^Univariate Cox-Regression analysis.

^**^Student *t*-test or Pearson χ^2^.

**Table 2:** Selected Variables by Multivariate Cox Proportional Hazards Regression Analysis and Prognostic Score.

Variables	Hazard Ratio	95% CI	*P*	Score
Age, year			**<0.001**	
<60	Reference			0
60–70	1.183	1.080–1.295	<0.001	0.9
>70	1.456	1.315–1.613	<0.001	2.4
Race			**0.007**	
White	Reference			0.4
Black	1.262	1.075–1.481	0.004	1.9
Other	0.916	0.764–1.098	0.344	0
Histology			**0.002**	
Adenocarcinoma	Reference			0
Squamous cell carcinoma	1.174	1.060–1.299	0.002	1.0
Tumor site			**0.002**	
Upper	Reference			2.6
Middle	1.013	0.795–1.291	0.914	2.7
Lower	0.842	0.664–1.067	0.154	1.4
Overlapping	0.910	0.666–1.245	0.556	1.9
Unknown (X)	0.638	0.457–0.890	0.008	0
Tumor size (cm)			**<0.001**	
≤3	Reference			0
≤5	1.247	1.116–1.393	<0.001	1.3
*>*5	1.359	1.209–1.527	<0.001	1.9
Unknown (X)	1.151	1.027–1.289	0.016	0.8
Grade			**<0.001**	
Well differentiated	Reference			0
Moderately differentiated	1.311	1.062–1.617	0.012	1.4
Poorly or Undifferentiated	1.683	1.367–2.072	<0.001	3.2
Unknown (X)	1.024	0.799–1.313	0.853	0.1
Pathologic T category			**<0.001**	
T1	Reference			0
T2	1.477	1.304–1.673	<0.001	2.3
T3	1.802	1.615–2.011	<0.001	3.8
T4	1.976	1.680–2.325	<0.001	4.7
Pathologic N category			**<0.001**	
N0	Reference			0
N1	1.989	1.802–2.195	<0.001	2.8
N2	3.038	2.682–3.441	<0.001	5.7
N3	4.582	3.876–5.416	<0.001	10
No. of nodes retrieved			**<0.001**	
0–9	Reference			3.7
10–14	0.763	0.687–0.847	<0.001	1.7
15–24	0.615	0.550–0.687	<0.001	0.4
>25	0.557	0.477–0.651	<0.001	0
