# Supplementary material for: Clinical Nomogram for Predicting Survival of Esophageal Cancer Patients after Esophagectomy
Source: Sci Rep. 2016 May 24;6:26684. doi: 10.1038/srep26684 (PMC4877645; doi:10.1038/srep26684)
Supplement: Supplementary Information [file srep26684-s1.pdf]

Clinical Nomogram for Predicting Survival of Esophageal Cancer Patients after Esophagectomy

Jinlin Cao, Ping Yuan, Luming Wang, Yiqing Wang, Honghai Ma, Xiaoshuai Yuan, Wang Lv & Jian Hu

Supplementary S1. The program selection codes for the Surveillance, Epidemiology, and End Results (SEER) database queries:

{Race, Sex, Year Dx, Registry, County.Year of diagnosis} = '1988','1989','1990','1991','1992','1993','1994','1995','1996','1997','1998','1999','2000','2001','2002','2003','2004','2005','2006','2007'

AND {Race and Age (case data only).Age recode with single ages and 85+} = '18 years','19 years','20 years','21 years','22 years','23 years','24 years','25 years','26 years','27 years','28 years','29 years','30 years','31 years','32 years','33 years','34 years','35 years','36 years','37 years','38 years','39 years','40 years','41 years','42 years','43 years','44 years','45 years','46 years','47 years','48 years','49 years','50 years','51 years','52 years','53 years','54 years','55 years','56 years','57 years','58 years','59 years','60 years','61 years','62 years','63 years','64 years','65 years','66 years','67 years','68 years','69 years','70 years','71 years','72 years','73 years','74 years','75 years','76 years','77 years','78 years','79 years','80 years','81 years','82 years','83 years','84 years','85+ years'

AND {Site and Morphology.Primary Site} = 150-155,158-159

AND {Site and Morphology.Histologic Type ICD-O-3} = 8000-8576,8940-8950,8980-8981

AND {Site and Morphology.Diagnostic Confirmation} = 'Microscopically confirmed'

AND {Extent of Disease - CS.Regional nodes examined (1988+)} = 1-90

AND {Extent of Disease - CS.Regional nodes positive (1988+)} = 0-90

AND ({Extent of Disease - Historic.EOD 10 - extent (1988-2003)} = 10-80

OR {Extent of Disease - CS.CS extension (2004+)} = 100-800)

AND Not{Extent of Disease - CS.CS mets at dx (2004+)} = 1-99

AND {Multiple Primary Fields.Sequence number} = 'One primary only'

AND {Therapy.Radiation sequence with surgery} = 'No radiation and/or cancer-directed surgery','Radiation prior to surgery'

AND {Cause of Death (COD) and Follow-up.Type of follow-up expected} = 'Active follow-up'

AND {Cause of Death (COD) and Follow-up.Survival months} = 3-479
